# MISSISSIPPI’S BOLD PUBLIC HEALTH RESPONSE TO DEMENTIA IN RURAL AND TRIBAL AREAS

**DOI:** 10.1093/geroni/igae098.1227

**Published:** 2024-12-31

**Authors:** Kina White, Paulita Edwards-Childs

**Affiliations:** Mississippi State Department of Health, Jackson, Mississippi, United States; Mississippi State Department of Health, Jackson, Mississippi, United States

## Abstract

Individuals living in rural communities are at an increased risk for Alzheimer’s disease and related dementias (ADRD), which aligns with other complex place-based health disparities. ADRD represents a significant public health issue in Mississippi, with the state having the highest age-adjusted mortality rates due to ADRD in the nation. The burden of ADRD in Mississippi falls disproportionately on rural and minority populations, with the state having the highest percentage of Blacks/African Americans in the nation and all 82 counties being considered medically underserved due to a lack of physicians and healthcare facilities. Given that Alzheimer’s disease and related dementias are more common with advanced age, this may increasingly affect Indian Country with a higher health burden. Mississippi’s BOLD public health response to addressing ADRD includes a collective impact of key stakeholders and prevention strategies to emphasize a life course approach for improved health outcomes. The response encompasses strategies such as community education, caregiver support programs, early detection initiatives, and access to dementia-friendly services. Moreover, it emphasizes the importance of collaboration with tribal nations, leveraging local resources, and integrating traditional and healing practices into dementia care. Key examples of these strategies include establishing dementia-friendly congregations and communities, as well as training older adult volunteers as healthy aging champions to advocate for improved local policies that allow them to age in their places and communities.

